# Bioprospecting Antimicrobials from *Lactiplantibacillus plantarum*: Key Factors Underlying Its Probiotic Action

**DOI:** 10.3390/ijms222112076

**Published:** 2021-11-08

**Authors:** Maria Teresa Rocchetti, Pasquale Russo, Vittorio Capozzi, Djamel Drider, Giuseppe Spano, Daniela Fiocco

**Affiliations:** 1 Department of Clinical and Experimental Medicine, University of Foggia, 71122 Foggia, Italy; mariateresa.rocchetti@unifg.it; 2 Department of Agriculture Food Natural Science Engineering (DAFNE), University of Foggia, 71122 Foggia, Italy; pasquale.russo@unifg.it (P.R.); giuseppe.spano@unifg.it (G.S.); 3 Institute of Sciences of Food Production, National Research Council (CNR) of Italy, c/o CS-DAT, Via Michele Protano, 71122 Foggia, Italy; vittorio.capozzi@ispa.cnr.it; 4 UMR Transfrontalière BioEcoAgro1158, Univ. Lille, INRAE, Univ. Liège, UPJV, YNCREA, Univ. Artois, Univ. Littoral Côte d’Opale, ICV-Institut Charles Viollette, F-59000 Lille, France; djamel.drider@univ-lille.fr

**Keywords:** *Lactiplantibacillus plantarum*, lactic acid bacteria, probiotics, antibacterial extracellular compound, antiviral extracellular compound, probiosis, postbiotic, bacteriocin, plantaricin, organic acid, cell-free supernatant

## Abstract

*Lactiplantibacillus plantarum* (*L. plantarum*) is a well-studied and versatile species of lactobacilli. It is found in several niches, including human mucosal surfaces, and it is largely employed in the food industry and boasts a millenary tradition of safe use, sharing a long-lasting relationship with humans. *L. plantarum* is generally recognised as safe and exhibits a strong probiotic character, so that several strains are commercialised as health-promoting supplements and functional food products. For these reasons, *L. plantarum* represents a valuable model to gain insight into the nature and mechanisms of antimicrobials as key factors underlying the probiotic action of health-promoting microbes. Probiotic antimicrobials can inhibit the growth of pathogens in the gut ensuring the intestinal homeostasis and contributing to the host health. Furthermore, they may be attractive alternatives to conventional antibiotics, holding potential in several biomedical applications. The aim of this review is to investigate the most relevant papers published in the last ten years, bioprospecting the antimicrobial activity of characterised probiotic *L. plantarum* strains. Specifically, it focuses on the different chemical nature, the action spectra and the mechanisms underlying the bioactivity of their antibacterial and antiviral agents. Emerging trends in postbiotics, some in vivo applications of *L. plantarum* antimicrobials, including strengths and limitations of their therapeutic potential, are addressed and discussed.

## 1. Introduction

*Lactiplantibacillus plantarum* (*L. plantarum*), previously known as *Lactobacillus plantarum*, is a versatile species of lactobacilli. This subgroup of lactic acid bacteria (LAB) encompasses prokaryotes present in a range of diverse environments, including the gastrointestinal tract (GIT) of mammals, vaginal mucosa, food matrices, soil, and vegetable-associated niches. In this light, *L. plantarum*, in reason of a flexible behaviour, is found in association with all these niches (in several cases, with a dominant character) and is largely employed as starter cultures in the food industry. Selected *L. plantarum* strains are used to promote and/or carry-over fermentation processes that are functional to food production addressed to human consumption [1]. For its capacity to ferment and preserve food, enhancing its sensory properties and nutritional value, *L. plantarum* boasts a millenary tradition of safe use and thus shares a long-lasting relationship with humans. Moreover, like most lactobacilli, this species is generally recognized as safe (GRAS) and has been included by EFSA in the list of microorganisms with Qualified Presumption of Safety (QPS) [2]. More recently, the connection between humans and *L. plantarum* has been further strengthened by experimental evidences that highlight the probiotic character of several strains [3], some of which are commercialised as health-promoting supplements and functional food products [4]. For all the properties mentioned above, this species represents a good model to deepen the nature and mechanisms of antimicrobials as key factors related to probiotic action of lactobacilli. The status of probiotics connotes microorganisms endowed with the ability to confer health benefits on the host upon ingestion in adequate amounts [5]; this depends on a set of microbial properties which include, among others, the aptitude to survive to the harsh conditions imposed by the human GIT, the capacity to colonise, at least transiently, the intestinal mucosa, to reinforce gut barrier function, to preserve the balance of the gut microbiota and prevent dysbiosis, to stimulate immune responses by interacting with host defence cells, to support digestive functions, and to synthesise vitamins, short-chain fatty acids and/or bioactive molecules that may be helpful for the host [6,7,8,9]. Likewise, the debated term “postbiotic” has been emerging recently to indicate inanimated microbial cells, cellular components and/or metabolites that promote the observed health benefit [10,11,12].

In this regard, the production of antimicrobials is associated with some important probiotic properties, as well as relevant protechnological features of LAB in food and beverage applications [13,14,15,16]. Indeed, the antimicrobial compounds derived from the lactobacilli colonising the gut can keep under control the growth of potential pathogens and opportunistic species, thus playing a relevant part in the complex net of relationships that ensure the homeostasis of the intestinal ecosystem, and contributing to the host health [17,18,19]. Moreover, by antagonising common food spoilage/contaminating microbes, antimicrobials from starter lactobacilli ensure food safety and prolonged bio-preservation [20,21,22].

**Figure 1:**
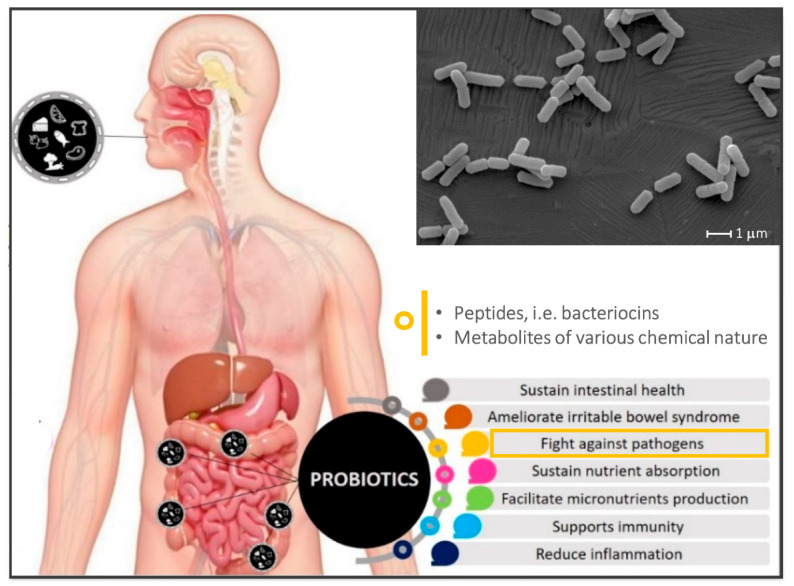
Conceptualisation of the intake of probiotic-containing foods/beverages and of the most investigated beneficial properties exerted by probiotics, with a specific emphasis on the antimicrobial chemicals produced by lactobacilli. The indicated probiotic mechanisms are putative, not always sufficiently proven, and may depend on bacterial number, host gut microbiome composition and the specific probiotic strain. Image partially reproduced from Pop et al. [30] (copyright 2020 MDPI). In grey, an original picture of *Lactiplantibacillus plantarum* WCFS1 cells imaged by scanning electron microscope (SEM).

The main antimicrobial chemicals produced by lactobacilli comprise (*i*) ribosomally synthesised peptides, i.e., bacteriocins [23] and (*ii*) metabolic by-products of various chemical nature, such as hydrogen peroxide (H_2_O_2_) [24], lactic acid and other organic acids [25], phenolic compounds [26,27], etc. While bacteriocins typically exhibit selective and target-specific antagonistic activity [23], the latter group comprise molecules that generally act rather aspecifically in inhibiting the growth of competitor species (Figure 1). Taking into account the worldwide healthcare emergency of the increasing (multi) drug resistance of infectious agents, the antimicrobials produced by probiotic lactobacilli, especially bacteriocins, may be suitable alternatives to conventional antibiotics and thus hold great potential in several biomedical applications [18,28,29].

In this review, we aim to survey some of the most relevant and recent papers, among those published in the last decade, bioprospecting the antimicrobial activity of characterised probiotic *L. plantarum* strains. Specifically, we focus on the different chemical nature of the antibacterial and antiviral agents produced, their action spectra, and the mechanisms underlying their bioactivity. Furthermore, we highlight some in vivo applications of these compounds, discussing the limitations of their therapeutical potential.

## 2. Emerging Trends in Probiosis, Postbiotics and Antimicrobials

**Figure 2:**
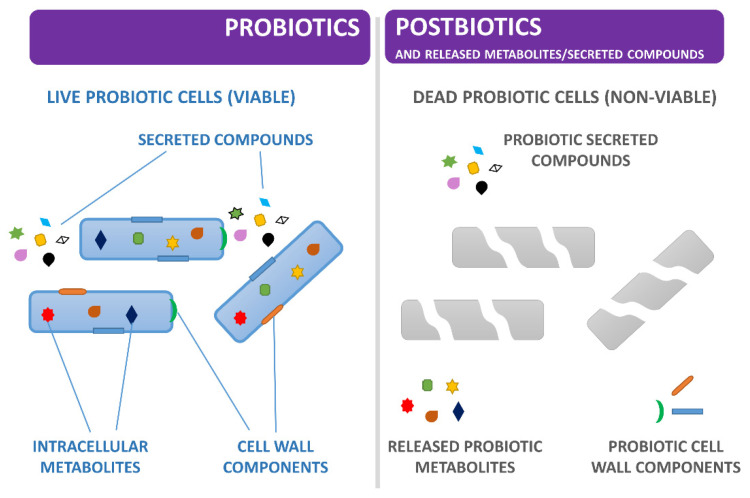
Graphic schematisation of the concepts of probiotic (on the left), postbiotic and released metabolites/secreted compounds (on the right). This review focuses on *L. plantarum* secreted compounds (i.e., CFS or isolated compounds from CFS) with antimicrobial activity.

While the terms probiotics, prebiotics, and synbiotics have been extensively defined in the last two decades and consensus documents have been provided by experts for each of them [5,31,32], the new concept of postbiotic is taking shape as an important microorganism-derived tool to promote health [10,11,12]. The need for a more precise terminology derived from observing the potential beneficial effect of non-viable microbial cells or effectors molecules contained, for example, in fer-mented foods (especially after prolonged storage) or in probiotic preparations (especially at the end of shelf life). The antimicrobial and antiviral activity exerted by beneficial microorganisms, such as *L. plantarum*, depends on a variety of molecules (organic acids, peptides, short-chain fatty acids (SCFA) and other antagonistic metabolites) that act with different action mechanisms and, sometimes, synergically produce the final bactericidal or bacteriostatic effects against the target microbes. Likewise, bacterial lysates have been shown to have some health benefits [33]. However, a precise distinction between the efficacy of non-viable beneficial bacteria, their growth products and their end-products on overall host health is not yet well defined, as each could have a beneficial role individually or in combination with the others. Very recently, postbiotics have been defined as a “preparation of inanimate microorganisms and/or their components that confers a health benefit on the hosts” [10], including in this concept the killed microbial cells with or without metabolites and excluding purified products (i.e., proteins, peptides, exopolysaccharides (EPS), SCFAs) [10]. The specific killed microorganism, the matrix and the inactivation method should be accurately indicated in the postbiotic definition. Until recently, the term postbiotics referred also to soluble factors secreted by live bacteria or released after bacterial lysis, also known simply as cell-free supernatants (CFS), i.e., quite heterogeneous mixtures including SCFAs, cellular enzymes, peptides, teichoic acids, peptidoglycan-derived muropeptides, EPS, cell surface proteins, vitamins, plasmalogens, and organic acids, which could have the same health beneficial effects of the strain that generated them [34]. However, a precise boundary line between what is currently defined postbiotic and what is not (i.e., CFS) is actually very difficult to delineate because some chemically synthesised compounds and/or metabolites might be present in both viable and not viable microbial cell preparations (Figure 2). Likewise, other scientists have pointed to some inconsistencies and ambiguities associated with the recently proposed re-definition of postbiotics [11]. Therefore, we wonder whether microbiologists should coin a new term to correctly define a probiotic bacterial CFS, which in most research work, is filtered to eliminate cells and cellular debris. Nonetheless, according to the International Scientific Association of Probiotics and Prebiotics (ISAPP), the term CFS is sufficiently defined as such and further definitions are not deemed as necessary [12].

In this context, not yet clearly defined, we gathered and reported in temporal order (Table 1) the data from the last decade on the chemical nature of compounds with antibacterial and/or antiviral activity as found in the CFS of probiotic *L. plantarum* strains. In addition, the investigated action mechanisms of the single antimicrobial substances are reported. We realise from these studies that, despite trying to understand and dissect the action mechanism of the single antimicrobial compound, the overall mechanisms of complex mixtures underlying food preservation and human/animal health are still far from being fully understood.

## 3. Nature and Mechanisms of *L. plantarum* Antimicrobials

Two modalities are basically involved in the antimicrobial action: (*i*) bacteriostatic, which means that the antimicrobial agent prevents the growth of target microbe, and (*ii*) bactericidal, which means that the agent kills the target cell. However, the exact microbiological bactericidal or bacteriostatic setting of bacterial-related agents may be influenced by growth conditions, bacterial density, duration of the test used, and measure of reduction in bacterial numbers [35].

The antimicrobials produced by lactobacilli are quite diverse and fall within two main chemical categories, proteinaceous and non-proteinaceous substances. Data reported in Table 1 and Table 2 show that the antimicrobial activity of *L. plantarum* is mainly exerted by bacteriocins (~60% of the reported strains) or partially characterised proteinaceous compounds, followed by organic acids or acidic conditions (i.e., acid CFS [25]), and biosurfactants (BS) such as glycoproteins and EPS. 

### 3.1. Bacteriocins

Bacteriocins are a heterogeneous group of ribosomally synthesised, gene-encoded peptides with specific antimicrobial activity towards a spectrum of target microbes, which can be narrow or broad depending on the producing strain [23]. The bacteriocins produced by *L. plantarum* spp. are referred to as plantaricins; generally, they are small, heat-stable, frequently very potent, being active at nanomolar concentrations, and exert their killing effect mostly through membrane permeabilization, through pore formation and subsequent leakage of cytoplasmic compounds. For their high antimicrobial versatility, they have been considered for use as bio-preservatives, antibiotic alternatives, health-promoting gut modulators and animal growth promoters [36]. 

As observed for other bacteriocins, the production of plantaricins is regulated through a quorum-sensing based network and seems to be switched on by specific signals that characterise the natural niche of the strain [37]. Stress conditions and co-culture with other specific inducing bacteria usually enhance plantaricin gene expression [38,39,40]. Likewise, the accumulation of signalling molecules, such as the PlnA peptide, outside the bacterial cells, can modulate bacteriocin production [40]. Intriguingly, transcriptomic and genomic studies in the reference strain *L. plantarum* WCFS1 pointed out that plantaricins synthesis may be triggered in vivo, during transit along the gut [41,42], thereby providing competitive advantages over other intestinal resident microbes, contributing to microbiota balance and possibly playing a role in the molecular interplay with the host immune system [43,44]. 

Like bacteriocins, even plantaricins can be roughly divided into two classes, which, in turn, include subclasses: Class I, containing post-translationally modified peptides, and Class II, containing predominantly unmodified peptides. Being amphiphilic, plantaricins interact electrostatically with the negatively charged bacterial cell surface (mainly constituted by phospholipids and lipopolysaccharides) by their positively charged group (cationic plantaricins) [45], while their hydrophobic portion passes through the membrane lipid bilayer of target bacteria. After internalisation, the peptides aggregate to form polymers or complexes, which create holes in the cell wall and the membrane, causing a change in permeability with consequent leakage and depletion of intracellular compounds (e.g., genetic and proteinaceous material), eventually leading to cell membrane lysis [46,47,48,49]. 

A few studies reported in Table 1 and Table 2 investigated the mechanism of action of plantaricins; among them, Kim and co-workers showed that plantaricins 3 and 5, from *L. plantarum* NIBR97, inhibited *S. Enteritidis* growth, causing its cellular lysis by damaging the membrane via pore formation [48]. Synthetic plantaricins 3 and 5 were further investigated for antiviral activity against GFP-labelled lentiviruses and, interestingly, scanning electron micrography (SEM) revealed that plantaricin 3 caused lentiviral lysis in human host cells through the collapse of their envelopes, while plantaricin 5 did not, implying two different antiviral mechanisms [50]. Tenea and co-workers investigated the mechanism by which Gt2 peptides and Cys5-4 peptides, from *L. plantarum* UTNGt2 and UTNCys5-4, respectively, target Gram-negative bacteria (Table 1). Gt2 and Cys5-4 peptides comprise, in turn, two or more post-translationally modified peptides forming one aggregate forming one functional inhibitory unit, which altered the cellular membrane permeability of *E. coli* and *Salmonella*, causing the leakage of cytoplasmic contents, followed by cellular death [50,51]. In a later study, the same authors investigated deeply the changes in *Salmonella* cells treated with Gt2 and Cys5-4 extracts, highlighting the occurrence of direct interaction between *Salmonella* genomic DNA and the peptides [46]. Such interaction has also been observed for antibacterial compounds from other species and was suggested to concur to the antimicrobial effects, i.e., by inducing DNA damages and genomic instability [52].

Data collected so far suggest that the action mode for plantaricins is usually bactericidal, through the induction of ion-selective pores in the target cell membrane, which causes the dissipation of intracellular ATP and depletion of the proton motive force, leakage of intracellular substances, followed by cell death. However, bacteriostatic effects have been reported for plantaricin W [53] and plantaricin LpU4 [54], as well as for other plantaricins previously studied [55,56]. In these cases, it has been suggested that plantaricin binds to target cell surface without reaching the specific receptors needed to achieve the killing effect [53]. In addition, certain *L. plantarum* strains produce more than one bacteriocin, either acting individually, e.g., bacteriocins F1 and F2 which exhibit different levels of efficacy against *Staphylococcus aureus* [57], or whose combination/synergic action results in higher antibacterial efficacy, e.g., plantaricin KL-1Y [58]. It is also worth mentioning that some plantaricins, such as KL-1Y [58], ZJ008 [59] and BM-1 [60], exert a bactericidal action without apparent cell lysis, whereas some, e.g., BM-1, can inhibit Gram-negative bacteria growth by bacteriostatic action, by influencing metabolic pathways and affecting the cell wall structure, eventually causing its collapse [61].

In addition to plantaricins, other *L. plantarum* proteinaceous compounds have been ascribed antibacterial activity. In most studies, such antimicrobials are generically referred to as bacteriocin-like peptides. For instance, the extracellular anti-staphylococcal protein fractions produced by a strain isolated from meat were recently identified as a couple of enzymes (i.e., transglycosylase and glyceraldehyde-3-phosphate dehydrogenase (GADPH)), which inhibit *S. aureus* growth through different mechanisms [62]. Extracellular transglycosylase binds to *S. aureus* peptidoglycan, thereby degrading the cell wall until cell lysis occurs; once the membrane has been damaged by transglycosylase, GADPH enters the cell and up-regulates *S. aureus* autolysis genes [62].

Considering the key role of the membrane surface charge and fluidity in the action mode of bacteriocins, it is easy to guess that the manipulation of these two bacterial properties may render the bacteriocins ineffective, resulting in bacteriocin resistance [63,64], or, on the other hand, in higher efficacy, which might be achieved for instance by bioengineering [65]. That being said, this represents an important mechanism of bacterial adaptation [66] that deserves to be studied and treated in depth separately, as it goes beyond the focus of this review.

### 3.2. Organic Acids

*L. plantarum* antimicrobial activity also often depends on the production and release of different types of organic acids (primarily, lactic and acetic acids, to follow tartaric, citric, malic, oxalic, and succinic acid) and on the associated pH lowering of the surrounding environment. Both factors concur to hinder the survival of acid-sensitive microorganisms [67]. Even short-chain fatty acids (SCFAs), e.g., butyric, propionic and valeric acids, and their modified derivatives have been ascribed antibacterial activity.

Since the growth of important food-spoilage and food-poisoning microorganisms is inhibited at low pH (<4), *L. plantarum*, which is one of the lactobacilli with the highest lactic acid production rate [25,68], can be added to many fermented foods as a natural preservative [69,70]. LAB, including *L. plantarum*, may be homofermentative or heterofermentative depending on the pathway used for glucose fermentation. Homofermentative bacteria produced more lactic acid through glycolysis compared to heterofermentative bacteria, which ferment glucose via the 6-phosphogluconate/phosphoketolase pathway [71]. Because different strains produce different types, amounts and combinations of organic acids, the resulting overall inhibitory actions are quite variable. There is much here to indicate that the antimicrobial mechanism resulting from pH acidification is species- and strain-specific [70,72]. The hydrophobic, undissociated form of the acid permeates the cell membrane and dissociates inside the target cell as a consequence of the intracellular neutral pH conditions, thereby acidifying the cytoplasm. The acid pH and the neutralisation of the electrochemical proton gradient stops various pH-dependent transport mechanisms causing bacteriostasis and eventually cell death [73,74]. The pH variation of the cytoplasmic environment depends on the specific pKa values of the organic acids produced by the *L. plantarum* strain, which explains the variability of their antimicrobial action [68]. Furthermore, by their chelating properties, organic acids can capture essential growth elements, such as iron [75].

Some modified acids have also been ascribed antibacterial activity. A derivative of propionic acid, i.e. 2-(2-1 mino-1-hydroxyethoxy) ethyl 2-methylpropanoate (LPB 102), was found to be the antimicrobial agent produced by *L. plantarum* NTU 102, with inhibitory action against *Vibrio parahaemolyticus*, a bacterium that is frequently associated with foodborne outbreaks of disease [76]. The authors attributed the inhibitory effects of LPB 102 to the suppression of specific *V. parahaemolyticus* genes that underlie its intrinsic resistance to various antimicrobial agents [77].

3-Phenyl lactic acid (PLA), a metabolite produced by some LAB from phenylalanine catabolism [78], is quite a new type of powerful and broad-spectrum antimicrobial compound that is active against both bacteria and fungi [79]. For its capacity to contrast food spoilage microorganisms, it is considered a valuable natural food preservative. Therefore, PLA biosynthesis, and strategies to increase its yield in starter LAB, have attracted much research effort [78,80]. PLA occurs in two enantiomers, L-PLA and D-PLA, whose difference in antibacterial capacity is still debated [81,82]. In a few pathogenic species, among those that are sensitive to PLA, this compound has been demonstrated to function by targeting the bacterial membrane [83,84], i.e., affecting its charge distribution and hydrophobic properties [85]. In *Listeria monocytogenes*, a mixture of the two isomers, obtained from a fermented vegetable *L. plantarum* isolate, was found to disrupt the cell membrane, and induce pore formation and leakage of intracellular material by interacting with cell membrane proteins [86]. Such mechanism of action has been recently confirmed also for the anti-*Salmonella* activity of PLA chemically characterised and purified from an infant faeces-isolated *L. plantarum* strain [26]. Intriguingly, the authors observed that, besides destroying the cell membrane, the purified compound was able to intercalate genomic DNA, suggesting a further mode of action for this molecule [26].

### 3.3. Biosurfactants

BS are amphipathic molecules with a hydrophilic head moiety and a hydrophobic tail, whose balance provides their surface activity [87]. In lactobacilli, BS are either extracellularly secreted or cell-bound components, and have been identified as chemically different molecules, including lipopeptides [88], glycopeptides [89], glycoproteins [90,91], glycolipids [92], phospholipids and polysaccharides [93]. Antimicrobials with BS properties usually exert a bacteriostatic action, and typically destabilise membranes and affect cell adherence, a key pathogenic feature, as it enhances colonisation ability by potential pathogens on both biotic (e.g., host mucosae) and abiotic surfaces (e.g., food, surgical instrument, implanted medical devices). BS from lactobacilli have been shown to inhibit foodborne pathogens [90,91,94], to possess antibiofilm [91,94] and antiadhesive [91,94] properties against fastidious or pathogenic microbes, as well as antiviral and anti-cancer activities [91], hence supporting their potential application in various fields, e.g., to contrast infections, particularly hospital-acquired infections [87,95], or to reduce microbial colonisation on food surfaces [96,97].

A few studies have characterised the action mechanism of proteinaceous BS from *L. plantarum* spp. Crude BS extracted from an *L. plantarum* cheese isolate were found to counteract, dose-dependently, biofilm formation of *S. aureus*, apparently by affecting the expressions of biofilm-related genes and by interfering with quorum-sensing signalling [94]. The BS produced by *L. plantarum* 60FHE was structurally characterised and identified as a mixture of glycoproteins, which exert antimicrobial activity against some foodborne pathogens (Table 1), possibly through penetration into the cell and by rupturing the membrane, leading to cell lysis [91]. Interestingly, the biosurfactant produced from this strain was also shown to be a potential anti-cancer agent [91].

Some *L. plantarum* exopolysaccharides (EPS) have BS-related antimicrobial properties. EPS, i.e., hydrophilic extracellular high-molecular-mass polymers, are produced by different LAB and exhibit high structural diversity in terms of sugar compositions, type of bonds between the repeating units, chain length, branching, and non-sugar modifications [87]. In *L. plantarum* the ability to synthesise EPS is a strain-specific trait and requires the presence of specific gene clusters encoding for regulatory factors and enzymes that enable biosynthesis and assembly of the sugar monomers and secretion of the polysaccharide [98]. In addition to the antimicrobial [99] and antibiofilm activities [100,101,102,103,104], *L. plantarum* EPS have been ascribed other properties that may impact the interaction with the host and can account for the health benefits provided by probiotic lactobacilli, such as immune-stimulating [98,105,106], antioxidant [107] and anti-cancer activities [108,109].

Generally, EPS from LAB, including *L. plantarum*, exert their antibacterial activity by interfering with the adhesion to surfaces and with cell adhesion/recognition mechanisms, thereby contrasting the formation of biofilm [99,103]. Biofilms are surface-associated, complex microbial communities, embedded in a self-synthesised polymeric matrix. These multicellular, three-dimensional structures develop thanks to inter-cellular signalling and through modulation of cell adhesion properties, and can confer to microbes a greater resistance to antibiotics [110]. Song and co-workers reported that EPS produced by *L. plantarum* 12 exert antibiofilm activity against *Shigella flexneri*, a foodborne enteric pathogen that can induce bacillary dysentery [101]. The authors found that the active form of its EPS (i. e., L-EPS) decreased polysaccharide production in the extracellular polymeric matrix of *S. flexneri* only by direct contact with the pathogen and without affecting its growth. L-EPS were hypothesised to disturb the signalling involved in biofilm formation and to interfere with the extracellular polymeric structures of the pathogen, which is crucial for maintaining the integrity of its biofilm [101]. Within biofilms, which can colonise the surfaces of medical equipment or food, microbial cells gain a greater resistance to disinfectants and conventional drugs, thus representing a serious global health concern [110]. Interestingly, EPS from a cheese-isolated *L. plantarum* strain were found both to inhibit *E. coli* biofilm formation and to reduce the activity of efflux pumps implicated in drug resistance [100]. The authors ascribed the antibiofilm effect to a decreased production of indole, i.e., a metabolite putatively involved in virulence and in the quorum-sensing systems sustaining biofilm development, and to a reduction of cell surface hydrophobicity, as observed in EPS-treated *E. coli* cells [100].

**Figure 3:**
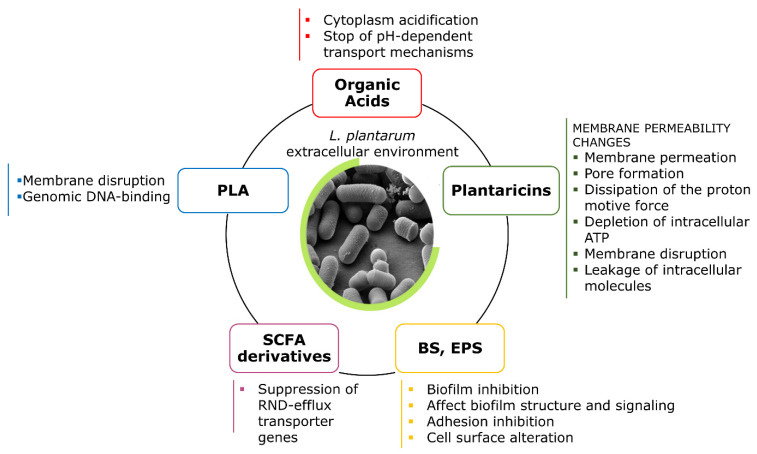
Different chemical nature of the principal antimicrobial agents produced by *L. plantarum* strains, and the suggested mechanisms underlying their antimicrobial effect. PLA: phenyl lactic acid; SCFA: short-chain fatty acids; BS: biosurfactants; EPS: exopolysaccharides.

Figure 3 sums up the different chemical nature of the principal antimicrobial agents produced by *L. plantarum* strains, and the suggested mechanisms underlying their antimicrobial effect.

What we are looking at reinforces the hypothesis that the antimicrobial action of a probiotic such as *L. plantarum* may not be due to a single molecule but to the synergic action of several molecules produced by the strain and released into the environment (i.e., growth media, food matrix or gut, according to application and niche of the strain). This is further supported by the observation that in most of the works on the isolation of the antimicrobial agents, CFS show a wider antimicrobial inhibition spectrum compared to the isolated antimicrobial agents [40,48,76,111]. The question is whether antimicrobial action of the isolated compound has been tested, or not, on all target pathogens used to test also the corresponding CFS. Namely, in most published papers, the antibacterial activity of CFS is usually tested on a broad spectrum of pathogenic bacteria, while the single CFS-derived antimicrobial compound is assayed on a single target. This can be a choice dictated by many reasons and does not necessarily mean that the isolated compound is active only against that specific target pathogen (i.e., the ones reported in Table 1). Then, perhaps, in view of applications in the food industry and human medicine, as bio-preservatives and bio-therapeutics, we should rethink our research of the single antibacterial agent (spending time and money in using complicated technologies) and focus more on the entire bacterial product (growth media), rather standardising times and methods of CFS collection and processing.

## 4. Antibacterial and Antiviral Spectrum of *L. plantarum* Extracellular Compounds

A key role of probiotics is that of preventing infections in the host, maintaining a healthy and balanced intestinal microbiota; likewise, microbes intended for use as starter and food preservatives should enhance food quality and safety, limiting contamination by fastidious and potentially dangerous microbial species. Therefore, a powerful, broad-spectrum antibacterial and antiviral activity against pathogens is strongly desirable, both as whole cells and as growth products/metabolites (intra and extracellular).

Table 1 shows that *L. plantarum* bacteriocins are effective against several pathogenic bacteria, including clinically relevant pathogens such as *L. monocytogenes* (~50% of the reported bacteriocins), a Gram-positive species which is widespread in nature (i.e., soil, vegetation, mammalian cells), robust, able to grow at refrigeration temperatures, and also recognised for a long time as a cause of human disease. Indeed, listeriosis can determine sepsis in immunocompromised patients, meningoencephalitis and febrile gastroenteritis [112]. The activity of these proteins against *L. monocytogenes,* sometimes regardless of pH [113], besides probiotic properties and safety of the producing strain, allow the development of novel bio-preservatives, with potential use in the food industry. At present, only two bacteriocins have been given the GRAS status, being approved for use as natural food preservatives, both produced by LAB (i.e., nisin, from *Lactococcus*, and pediocin PA-1D, from *Pediococcus* genus) [114]. Organic acids, mainly lactic acid, produced from *L. plantarum* extracted from kimchi [115] and bean [73], also showed to inhibit *L. monocytogenes* as well as, almost to the same extent, other pathogens, making the corresponding strains potentially useful as starter culture [73,115].

*L. monocytogenes*, along with *S. aureus* and *E. coli*, is also considered a foodborne pathogen, as these bacteria can produce enterotoxins in contaminated food. Several different plantaricins were found to be active also against *Listeria innocua*, the closely related but non-pathogenic *Listeria* species, often used in laboratories as a surrogate organism for a better understanding of the behaviour of the pathogen during food processing [116].

Quite a few bacteriocins from *L. plantarum* inhibit *S. aureus* (~60% of the studies reported in Table 1), one of the most common pathogens that can colonise intestine, skin tissues and perineal regions of the human host, causing severe infectious diseases, such as osteomyelitis, endocarditis, pneumonia, septicaemia, and health hazardous effects worldwide [117]. Indeed, *S. aureus* represents the most common microorganism causing infections in communities with very high economic burden at the social level; furthermore, it can develop considerable resistance towards conventional antimicrobial agents, with major prevalence of methicillin-resistant *S. aureus* (MRSA) and vancomycin-resistant *S. aureus* (VRSA) forms. For instance, MRSA accounted for 16% of necrotising soft tissue infections worldwide, although overall mortality is declining over the last ten years [117,118]. Examples of antimicrobials against these resistant forms comprise the CFS of *L. plantarum* strains extracted from sauerkraut [113] and from faecal microbiota [102], plantaricins LpU4 and ZJ008 from *L. plantarum* strains isolated from milk [54,59]. These extracellular compounds and their strains may represent an alternative bio-control strategy against skin infections. In addition, *L. plantarum* enzymes, i.e., transglycosylase and glyceraldehyde-3-phosphate dehydrogenase (GADPH), biosurfactants [90,94] and EPS [104] (in a dose-dependent manner), and, to a lesser extent, organic acids [73], all showed antagonistic activity against *S. aureus* [62].

Together with *L. monocytogenes* and *S. aureus*, *E. coli* is the most frequently inhibited by plantaricins (more than 50% of the *L. plantarum* strains/studies as reported in Table 1). This Gram-negative species represents the most prevalent commensal inhabitant of the human gastrointestinal tract, as well as one of the most common human and animal pathogens, being acknowledged as the causative agent of multiple clinical syndromes such as diarrhoeal diseases, meningitis and urinary tract infections [119]. In fact, although this bacterium is usually a benign gut commensal, some strains can acquire virulence, becoming able to cause diarrhoea in humans and other animals, and making *E. coli* one of the most widely studied etiologic agents worldwide [120]. Pathogenic *E. coli* forms causing diarrhoea have been classified into different pathotypes, including, among others, *enterotoxigenic E. coli* (ETEC), *enteropathogenic E. coli* (EPEC), Shiga toxin-producing *E. coli* (STEC), *enteroinvasive E. coli* (EIEC), *enteroaggregative E. coli* (EAEC), and diffusely adherent *E. coli* (DAEC) [120]. Interestingly, as shown in Table 1, ETEC and EPEC are inhibited mostly by organic acids and other unidentified extracellular compounds produced by several *L. plantarum* strains. Furthermore, EPS purified from a breast milk *L. plantarum* isolate demonstrated an excellent capacity to inhibit the adhesion of *E. coli* to epithelial human cells [107].

Plantaricins (especially Q7 [121], NC8 [122], Gt2 peptides and Cys5-4 peptide [46,51], IIA-1A5 [123], KL-1Y [58], ZJ316 [124], plantaricin 3, 5 [48]) are very active against *Salmonella* spp., a Gram-negative bacterium including pathotypes such as *Salmonella enterica* subspecies (*S. enteritidis*) and *Salmonella enterica* serotypes (*S. typhimurium*) (Table 1). Both can cause severe illnesses, ranging from gastroenteritis to typhoid (Typhi) and paratyphoid fever (Paratyphi), a global problem with more than 27 million cases worldwide each year [125,126]. Furthermore, salmonellosis, the contamination of food by Salmonella species, causes great harm to the livestock and poultry industries, thus, its prevention and control is of great importance to animal husbandry and public health [127]. Growth of *Salmonella* is also contrasted, through different mechanisms, including organic acids produced from several *L. plantarum* strains, EPS from *L. plantarum* YW32 and R315, and L-PLA from *L. plantarum* ZJ316 (Table 1).

*Bacillus cereus* is another common food contaminant with highly variable pathogenic potential ranging from strains that show little or no cytotoxic in vitro activity, to forms that are highly cytotoxic [128]. *B. cereus* can be responsible for two types of poisonings, depending on the toxin it produces, resulting in diarrhoea and emesis [129] (that in severe cases require hospitalisation and are sometimes fatal). *B. cereus* is also recognised as an aetiological agent of localised wound, eye and systemic infections [128]. Almost all plantaricins reported in Table 1 have been found active against *B. cereus* (corresponding to approximately 20% of the *L. plantarum* strains reported in Table 1) and in some cases, as for plantaricin GZ1-27, time- and dose-dependent activity was demonstrated [111]. Organic acids from *L. plantarum* S0/7 [73] and EPS from *L. plantarum* R315 [99] showed also an inhibitory activity against *B. cereus*. In addition, EPS from *L. plantarum* R315 were reported to inhibit *B. cereus*, other foodborne pathogens described above and *Cronobacter sakazakii*, an opportunistic Gram-negative bacterium that survives in very dry niches, and can contaminate food such as powdered infant milk, causing neonatal infections with high fatality rates [130,131].

*Pseudomonas aeruginosa* is another clinically relevant species, i.e., a Gram-negative, opportunistic pathogen with a high intrinsic resistance to a wide variety of antibiotics. *P. aeruginosa* is often found in medical equipment, such as inhalers, dialysis equipment, respirators, vaporisers, in toilets and sinks [132] and, consequently, it is the cause of several kinds of hospital-acquired infections, such as catheter-associated urinary tract infections [57], ventilator-associated pneumonia, gastrointestinal infections, dermatitis, skin infections, bacteraemia, bone and joint infections, and other infections, particularly in patients with severe burns and in immunocompromised subjects (i.e., suffering from cancer or AIDS) [133]. As shown in Table 1, the antimicrobial activity of *L. plantarum* against *P. aeruginosa* is mainly due to plantaricins. In addition, EPS produced by *L. plantarum* isolated from human breast milk showed a very strong inhibition for *P. aeruginosa*, higher compared to inhibition of other foodborne pathogens described so far [107].

Plantaricins produced from *L. plantarum* strains isolated from various niches also inhibited *Bacillus* spp. (*B. subtilis* and *B. anthracis*) [134,135,136,137], *Shigella* spp. [59,121,136,137,138], *Micrococcus luteus* [59,122,135,136,137,139,140], *Vibrio parahaemolyticus* [122,139,141], as well as *Clostridium* spp. (*C. butyricum*, *C. difficile* and *C. perfrigens*) [57,136], showing the potential for application in the food industry as well as therapeutics. Moreover, plantaricins isolated from *L. plantarum* strains isolated from yoghurt ‘dahi’, cheese and ‘dosa batter’ inhibited the growth and virulence properties of *Gardnerella vaginalis* [142,143], *Kocuria rhizophila* [144], and *Enterobacter cloacae* [135,139], respectively, demonstrating the potential application of *L. plantarum* spp. extracellular compounds for treating bacterial vaginosis [142], human infections [145], and obesity [146].

Researchers have focused mainly on the antibacterial and antifungal properties of *L. plantarum* compounds, whereas their antiviral action has been much neglected. Table 2 reports studies, mostly in vitro, documenting such activities. Plantaricins 3 and 5, produced by *L. plantarum* NIBR97, were found to exhibit antibacterial activities against a broad range of pathogens (Table 1), plus significant antiviral activities against the human pathogen influenza A virus (H3N2) (Table 2) [48]. Therefore, they were recently suggested as potential natural disinfectants, which might be an alternative to the chemical ones (alcohol- or chlorine-based preparations), for the disinfection of hands and surfaces in conditions of pandemics [48]. The proliferation of the influenza A virus was also found to be inhibited by proteinaceous compounds from *L. plantarum* LBP-K10 [147].

Other poorly defined extracellular metabolites from various *L. plantarum* strains (as reported in Table 3) could inhibit in vitro (i) *Echovirus*, enteroviruses isolates recovered from acute flaccid paralysis cases [148]; (ii) transmissible gastroenteritis virus *(TGEV)*, which causes many gastrointestinal infections in piglets, characterised by diarrhoea and high mortality [149]; (iii) enterovirus Coxsackievirus *B4,* a challenging virus, infections of which have been linked to the onset of type 1 diabetes [150]; (iv) porcine epidemic diarrhoea virus (*PEDV*), a coronavirus responsible of one of the highly contagious viral diseases in the pig industry, causing severe (sometimes fatal) diarrhoea in piglets [151]; (v) human rotavirus, i.e., the causative agent of severe diarrhoea in newborns and children worldwide [152]. Notably, the addition of prebiotics, such as those derived from microalgae, was reported to enhance both viability and antiviral effects of probiotics, as was observed for *L. plantarum* ATCC LP299v [153]. The anti-rotavirus action could account for beneficial effects of probiotics (mainly *bifidobacteria* and *lactobacilli*, including *L. plantarum*) in preventing enteric infections and alleviating diarrhoea symptoms [154]. In fact, dietary intake of *L. plantarum* LRCC5310, whose EPS were shown to inhibit the growth of rotavirus in vitro and in mice model [152], was subsequently found to be effective and safe in patients with rotaviral enteritis [154].

The spreading drug resistance in all cited microbial pathogens makes it difficult to treat and eradicate them and represents a severe problem for public health, requiring the development of alternative antimicrobial strategies. In this regard, the characterisation of antibacterial extracellular compounds produced by *L. plantarum* species opens new horizons in managing drug resistance. Particularly, bacteriocins might help to tackle antibiotic-resistant bacterial pathogens, a phenomenon that has become a worldwide threat, considering that the number of deaths per year due to antimicrobial resistance is predicted to exceed that of people who die from cancer [155,156].

## 5. In Vivo Studies on *L. plantarum* Strains Whose Antibacterial Activity Was Earlier Characterised In Vitro

In the medical field, *L. plantarum* is being investigated for an increasing number of applications such as: healing of skin wounds and burn infections [102,157,158]; treatment of mucosal infections [159,160]; protection from environmental mutagens [161,162]; amelioration of acute and chronic GIT infections [163,164], gut inflammatory disorders and urinary tract infections [165]; cholesterol level-lowering properties [166]; and beneficial effects on obesity [167], diabetes [168], colon cancer [169] and cognitive impairments [170]. Such broad range of possible utilisations reflects the genomic diversity of *L. plantarum*, which entails its large phenotypic diversity, versatility and flexibility [171].

This paragraph briefly discusses only the studies (reported in Table 3) which combine both in vitro and in vivo approaches to assess the antimicrobial ability of *L. plantarum* strains/compounds and those that investigate in vivo *L. plantarum* strains and/or related metabolites, whose antimicrobial effectiveness was earlier characterised in vitro (i.e., reported in Table 1). Noticeably, the number of in vitro studies (Table 1) far exceeds those where such strains and/or their isolated compounds are tested through in vivo experiments (Table 3). Most likely, we have to consider that, for example, the path from the discovery of the antibacterial activity of bacteriocins (by in vitro models) to their application as therapeutic agents is long, and involves many crucial steps to advance into clinical trials, such as the use of animal models and studies on toxicity and biosafety in vivo [172].

Moreover, it is worth highlighting that, in several of the studies reported in Table 3, it is not possible to conclude whether the observed effect is caused by antimicrobials alone or by a combination of factors, e.g., by antimicrobials or the *L. plantarum* strain per se, by nutrient competition, or through the induction of host antimicrobial proteins.

A few preclinical studies have prospected the use of *L. plantarum*-derived bacteriocins as a promising tool to control post-operative infections. In two independent studies, systemic treatments based on the intravenous injection of *L. plantarum* bacteriocins were shown to contrast *S. aureus* bone fracture-associated infections. Using two different animal models (i.e., rabbit and mice), and upon bacteriocin treatment, the authors observed a reduction of pathogen biofilm and a decreased serum level of pro-inflammatory markers [173,174]. Coherent findings were also reported by similar investigations using bacteriocins from other lactobacilli [175]. The above-mentioned preclinical studies rely on systemic administration of the isolated antimicrobials. However, a higher therapeutic effect could be probably achieved by topic application of the antimicrobial compounds, i.e., by their direct administration at the host surface, such as oral cavity, gut, skin, and urogenital mucosa, as was also recommended for postbiotics [10]. For instance, antimicrobials could be helpful to promote healing and prevent infection at wounds, ulcers and burn sites [176,177,178]. However, so far, most of such studies concerning *L. plantarum* cell extracts and/or secreted metabolites have been carried out mainly in vitro [158].

Recently, an elegant study in mice demonstrated the key role of bacteriocin for the anti-infective action of probiotics in the host gut [179]. This study provides clear molecular evidence that protective and anti-listerial effects of a bacteriocin-producing *L. plantarum* strain depend just on its ability to synthesise the bacteriocin, in situ, i.e., within the gut. The authors found that oral intake of bacteriocin-negative mutants of *L. plantarum* 423 failed to exclude *L. monocytogenes* from the gastrointestinal tract of mice, while administration of wild type *L. plantarum* could not inhibit gut colonisation by recombinant *L. monocytogenes* strains expressing the plantaricin immunity proteins.

Other animal studies indicate that oral intake of *L. plantarum* strains endowed with antimicrobial properties can contribute to (preserve or restore) gut microbiota balance and thus support future approaches to combat enteric infections and associated GIT inflammations. For instance, Choi et al. observed antiobesity effects in mice fed with a *L. plantarum* strain isolated from Kimchi (a traditional Korean fermented food), and ascribed these to its in vitro inhibitory activity against obesity-inducing bacteria (i.e., *Enterobacter cloacae*) [146]. Likewise, a plantaricin-like heat-stable antimicrobial was isolated and partially characterised by a food isolate *L. plantarum* and oral administration of such strain resulted in a healthy recovery of mice infected by *S. Typhimurium* [180]. Moreover, a *L. plantarum* strain, whose supernatants inhibited *Helicobacter pylori* growth in vitro, could attenuate *H. pylori*-induced gastric inflammation in rats [181].

*L. plantarum* with antimicrobial properties may have applications even in veterinary science and livestock industry, e.g., for the management of seafood farming, as studied by Chomwong and his co-workers [182]. These authors investigated the antipathogenic effect of *L. plantarum* SGLAB01, a strain isolated from the gut of shrimp. Dietary supplementation of such strain was found to enhance resistance to infections by *Vibrio parahaemolyticus,* which is responsible for acute hepatopancreatic necrosis, a disease implying troubles in intensive shrimp aquaculture [182]. This work also demonstrates the successful use of host-derived probiotics, i.e., microbes isolated from the digestive tract of the animal that is itself the target of the microbe-based therapeutic strategy. Such an approach reflects an increasing and up-to-date awareness that the animal and, specifically, human gut provides rich and as yet poorly explored reservoirs of potentially curative microorganisms, i.e., the so-called *next-generation probiotics* [183]. In very recent preclinical and clinical studies, (alterations of) some of these next-generation probiotics, identified as members of the human gut microbiota, have been associated with diverse diseases and hence have been indicated to own promising therapeutic potentials, e.g., for the treatment of diabetes and other endocrine/metabolism-related diseases [184].

## 6. Conclusions

As supported by the papers discussed in the present review, it is undoubtful that antimicrobial properties are of utmost relevance for the health-promoting effect of probiotics. Although several in vitro studies suggest a vast therapeutic potential for the antimicrobials from *L. plantarum*, to date, only a handful of investigations have explored the feasibility of their applications in vivo. Even if *L. plantarum* antimicrobials were found to be active against a broad spectrum of pathogens, just a few of them have been subsequently proven to prevent or ameliorate disease phenotypes in animal models. The numerical discrepancy between in vitro studies on *L. plantarum* antimicrobials and in vivo investigations might depend on the demanding organisation and management of the protocols required for experiments on animals, and then, in humans. A limiting factor may also be the isolation or the synthesis of adequate amounts of antimicrobial compounds to conduct in vivo investigations. In addition, comparative studies to assess the effectiveness of the isolated antimicrobial compound and the corresponding strain, in the form of viable cells, would be very useful and could indicate the way forward for probiotic research. Therapies based on probiotics, e.g., *L. plantarum* and probiotic-derived factors, have a high potential for the treatment of disorders, especially infections and gastrointestinal tract (GIT) diseases, and much research is still needed to define and consolidate it.

## Figures and Tables

**Table 1:** Probiotic *L. plantarum* strains with documented in vitro antibacterial activity.

Isolation Niche	Strain Name	Type of Antimicrobial	Investigated Action Mechanism	Strong Antimicrobial Activity/ Inhibited Bacterial Species	Reference
Fermented cocoa	Lp ^1^ 03, Lp 289, Lp 291	Organic acid (lactic acid)	n.i. ^2^	*Gardnerella vaginalis, Neisseria gonorrhoeae*	das Neves Selis N, 2021
Yoghurt fermented by koumiss	Lp RUB1	Class II bacteriocin	n.i.	*Bacillus cereus ATCC 14579*	Wu A, 2021
Cheese	Lp 60FHE	Biosurfactant: glycoprotein	Cell membrane lysis	*Staphylococcus epidermidis ATCC 12228, Microcccus luteus ATCC 10240, Escherichia coli ATCC10536, Pseudomonas aeruginosa ATCC 9027, Salmonella typhimurium, Enterobacter aerogenes 9805, Serratia marcescens 98027, Staphylococcus aureus ATCC 29737, Bacillus. pumilis ATCC 14884, Bacillus subtilis*	Sakr AE, 2021
Ghanaian traditionally fermented cow milk	Lp NL27 Lp PA27	CFS ^3^	n.i.	*E. coli, S. Typhimurium*	Motey GA, 2021
Indonesian traditional fermented meat	Lp S34	Plantaricin S34	n.i.	*Enteropathogenic E. coli (EPEC) K1.1., S. aureus, Salmonella typhosa, S typhimurium, Proteus* sp.	Ahaddin AY, 2021
Nem ‘chua’ (vietnamese sausage)	Lp B21	Plantacyclin B21AG	Deduced by comparisons with other circular bacteriocins using multiple sequence alignment: insertion into the phospholipid bilayer of the target cell membrane	*Clostridium perfringens 52/6-1, Listeria monocytogenes 192/1-2 ACM 3173*	Golneshin A, 2020
Kimchi	Lp NIBR97	Plantaricin 3, 5	Cellular lysis via pore formation in bacterial membranes by cellular penetrating peptides	*Salmonella enterica Serovar Enteritidis*	Kim SW, 2020
Sauerkraut	Lp SF9C	Plantaricin	n.i.	*L. monocytogenes* ATCC^®^ *19111™, S. aureus 3048, S. enterica serovar Typhimurium FP1, E. coli 3014*	Butorac K, 020
Kimchi	Lp EM	Plantaricin and bovicin	n.i.	*Vibrio parahaemolyticus* ATCC 17802, *P. aeruginosa*, *S. enterica* serovar Typhi, *B. cereus*	Kim E, 2020 Choi EA, 2015
Yoghurt	Lp ZX27	Plantaricin CFS	n.i. Reduction in *G. vaginalis* biofilm formation and preformed biofilm; suppressing the expression of genes related to *G. vaginalis* pathogenicity	*E. coli, G. vaginalis*	Qian Z, 2020 Qian Z, 2021
Intestines of a turbot	Lp-12	EPSs ^4^	Inhibition of biofilm formation	*Shigella flexneri*	Song Y, 2020
‘Dahi’, a fermented milk product	Lp DHCU70, Lp DKP1	NC8 type of bacteriocin	Inhibition of cell wall biosynthesis	*Kocuria rhizophila*	Goel A, 2020
Infant’s feces	Lp zrx03	Bacteriocin	n.i.	*S. aureus ATCC 25923, E. coli JM109 ATCC 67387, B. subtilis CICC 10002, Bacillus anthracis CICC 20443, Salmonella CMCC 541*	Lei S, 2020
Human oral cavities	Lp 108	CFS	Inhibited growth and biofilm formation by preventing microbial coaggregation; inhibit the adhesion of *Streptococcus mutans* and *Candida albicans* to solid surfaces	*Streptococcus mutans UA159*	Srivastava N, 2020
Slovak raw sheep milk cheese	Lp L5, L19, L20, and L22	Partially purified bacteriocins	n.i.	*L. monocytogenes, S. aureus*	Vataščinová T, 2020
Weaned piglet faeces	Lp ZA3	lactic acid and acetic acid	n.i.	Enterotoxigenic *E. coli (ETEC) K88*	Wang W, 2020
Stool human samples	Lp 69.1	CFS	n.i.	*ETEC* and Enteroaggregative *E. coli (EAEC)*	Pazhoohan M, 2020
Faeces of healthy infants	Lp 34-5	CFS (pH acid)	n.i	*S. flexneri* ATCC *12022, ETEC H10407 enteropathogenic bacteria*	Pazhoohan M, 2020
Wild-type fruits of *Theobroma grandiflorum* (white coffee), and *Malus* sp.	Lp UTNGt2, Lp UTNCys5-4	Gt2 peptides, Cys5-4 peptide	Cell membrane disruption and leaking of cytoplasmic β-galactosidase, RNA and DNA molecules. Binding and interacting with pathogen genomic DNA	*S. enterica subsp. enterica* ATCC *51741*, *E. coli* ATCC *25922*, *Shigella sonnei* ATCC *25931*	Tenea GN, 2020, 2019a, 2019b
Faeces of infants	Lp N20	Organic acid	n.i.	*Yersinia enterocolitica ATCC 23715, S. flexneri ATCC 12022, S.enterica ATCC 9270, enteropathogenic E. coli (EPEC) ATCC 43887*	Jomehzadeh N, 2020
Kimchi	Lp KU200656	CFS	Downregulation of the expression of pathogen’s biofilm-related genes	*S. aureus ATCC 6538, L. monocytogenes ATCC 15313, E. coli ATCC 25922*	Lee JE, 2020
Honey	Lp H46, H47, and H59	CFS	n.i	*S. flexneri ATCC 12022, S. aureus ATCC 25923, S. enteritidis F17, EPEC E2348/69, E. coli O157 H7 EDL 933, B. cereus D14*	Lashani E, 2020
Faeces of healthy infants	Lp ZJ316	L-PLA ^5^ Plantaricin ZJ316 Plantaricin NC8	Membrane destruction and DNA binding n.i. Cell membrane permeabilization and disruption	*S.enterica subsp. enterica ATCC 14028.* *L. monocytogenes, Listeria welshimeri, E. coli JM109, Pseudomonas putida ATCC 23288, S. enterica ZJJK18.* *S. enterica, S. typhimurium, Salmonella paratyphi-A, S. paratyphi-B, Micrococcus luteus CGMCC 1.193, V. parahaemolyticus, Staphylococcus epidermidis*	Zhou Q, 2020 Chen L, 2018 Jiang H, 2018 Jiang H, 2016
Sauerkraut	Lp NRRL B-4496	Proteinaceous compound CFS (acid)	n.i	*L. monocytogenes* *Methicillin resistant S. aureus (MRSA), L. monocytogenes, E. coli*	Arrioja-Bretón D, 2020
Pork minced meat	Lp USM8613	Transglycosylase and glyceraldehyde-3-phosphate dehydrogenase (GADPH)	Cell wall-mediated killing mechanism; GADPH penetrates into *S. aureus* cells, inducing the overexpression of autolysis regulators	*S. aureus*	Ong JS, 2019
Vaginal microbiota	Lp GF011	CFS (acid pH)	n.i.	Uropathogens: *S. aureus* sp. *GF01*, *P. aeruginosa GF01*, *Klebsiella* sp. *GF01*	ADEOSHUN FG, 2019
Yoghurt, Fermentation of millet and urum	Lp P1, S11, and M7	Organic acid (lactic, acetic, tartaric and malic acids)	n.i.	*E. coli and S. typhimurium*	Hu CH, 2019
Kimchi	Lp SPC-SNU 72-2	Organic acid	n.i	*E. coli O157, L. monocytogenes, S. typhimurium, H. pylori*	Park DM, 2019
Tarkhineh human faeces Lighvan cheese	Lp PT10 Lp PF11 Lp PL4	Bacteriocins	n.i	*E. coli O157:H7, S. typhimurium*	Joghataei M, 2019
Kimchi	Lp LMT1-48	SCFA ^6^ (hypothesised)	n.i	*E. cloacae*	Choi WJ, 2019
Sorghum beer Fruits and vegetables from Pakistan	Lp 423 Lp AS-4, AS-14	Plantaricin 423	n.i n.i.	*L. monocytogenes* *Listeria innocua, E. coli EC10, L. monocytogenes DPC 6179*	van Zyl WF, 2019 Manzoor A, 2019
NIQCH (Brazil)	Lp ATCC 8014	CFS (pH acid) Bacteriocin Bacteriocin	n.i n.i Growth inhibitory activity against planktonic cells; inhibition of biofilm formation	*Clostridium butyricum, Clostridium difficile, C. perfringens* *S. aureus, S. marcenses*	Monteiro CRM, 2019 Fu T, 2017 Shahandashti RV, 2016
Artisanal milk cheese	Lp 27172	Biosurfactants	Inhibits adhesion and biofilm formation by interfering with AI-2 signalling molecules and reducing expression of biofilm-related genes	*S. aureus CMCC 26003*	Yan X, 2019
Pineapple	Lp NRIC 149	Plantaricin 149	Carpet-like model of interaction with Gram + membrane	*Listeria and Staphylococcus genera*	Kumagai PS, 2019
Faeces of healthy humans	Lp PBS067	Plantaricin P1053	n.i.	*S. aureus, E. coli*	De Giani A, 2019
Koumiss	Lp MXG-68	Plantaricin MXG-68	Bactericidal mode of action	*L. monocytogenes ATCC 15313, B. cereus ATCC 11788, E. coli ATCC 25922, and S. typhimurium ATCC 14028.*	Man L, 2019
MTCC	Lp subsp. argentoratensis SJ33	Bacteriocin F1 and F2	Bactericidal activity on *S. aureus* by membrane pore formation and leakage of cellular contents; antibiofilm activity for *P. aeruginosa*	*P. aeruginosa and S. aureus, Aeromonas hydrophila, Clostridium sporogenes, C. perfringens, E. coli, Klebsiella pneumoniae*	Mohapatra AR, 2019
Faeces of breastfed infant	Lp F-10	CFS (acid pH), EPSs	Reduced quorum-sensing signals needed for biofilm formation, CFS might modify the target surface, causing a reduction or inhibition of irreversible attachment of the biofilm-forming bacteria that prevent biofilm formation	*P. aeruginosa PAO1/ATCC 27853, MRSA ATCC 43300*	Onbas T, 2019
Papaya	Lp ST16Pa	Bacteriocin ST16Pa	Cell lysis and enzymes leakage	*L. innocua, Latilactobacillus sakei, Enterococcus faecalis*	Sabo SS, 2019; Todorov SV, 2011
Cabbage pickles	Lp NTU 102	LPB102 ^7^	Suppression of resistance nodulation cell division (RND)-type efflux transporter genes	*V. parahaemolyticus, Cronobacter sakazakii*	Lin T, 2019
Yoghurt	Lp DM 69	Protein (MW 12.0 kDa) Proteinaceous compound	Inhibited adhesion and invasion of *S. enterica* into colon cells	*S. enterica subsp. enterica ATCC 35640* *B. cereus ATCC 10702, S. aureus subsp. aureus ATCC 29213, S. aureus MTCC 902, P. aeruginosa MTCC 741, Klebsiella pneumonia MTCC 109*	Mohanty DP, 2019 Mohanty DP, 2016
Fish	Lp LPL-1	Bacteriocin LPL-1	Increases membrane permeability, induces collapse of proton motive force, inhibits expression of genes related to virulence factors, biofilm formation factors, and RNA polymerase sigma factor	*L. monocytogenes 54002*	Wang Y, 2019 and 2018
Ricotta cheese	Lp L899	EPSs	Inhibition of biofilm and efflux pumps	*E. coli ATCC 35218*	Mahdhi A, 2018
Salted and fermented shrimp	Lp FB003	CFS	n.i.	*L. monocytogenes, S. aureus, Salmonella enterica serotype Choleraesuis, V. parahaemolyticus*	Le B, 2018
Shrimp gut	Lp SGLAB01	CFS	Modulation of the host proPO ^8^ system	*Aerococcus viridans, Vibrio harveyi, S. aureus, Bacillus megaterium, Bacillus subtilis, E. coli, V. parahaemolyticus*	Chomwong S, 2018
Yak cheese	LP SLG1	Plantaricin SLG1	Bactericidal mode of action, it damages cell membrane and induces the release of cytoplasmic components	*B. subtilis, B. cereus, B. megaterium, M. luteus, Brochothrix thermosphacta, C. butyricum, S. aureus, L. innocua, L. monocytogenes, E. coli, P. aeruginosa, Enterobacter cloacae and Salmonella paratyphi b*	Pei J, 2018
Fermented chinese milk	Lp J23	Bacteriocin Lac-B23	n.i.	*L. monocytogenes*	Zhang J, 2018
Dong-nationality kipper	Lp GZ1-27	Plantaricin GZ1-27	Increased cell membrane permeability, triggered K+ leakage and pore formation, damaged cell membrane integrity, reduced expression of genes related to cytotoxin production, peptidoglycan synthesis, and cell division	*B. cereus*	Du H, 2018
Sai krok e-san mu	Lp SKI19	BLIS	n.i.	*L. monocytogenes DMST 17303, B. cereus DMST 5040, C. perfringens DMST 1663, S. aureus DMST 8840, E. coli DMST 4212, S. Typhimurium DMST 15674, S. enteritidis DMST 15676*	Botthoulath V, 2018
Cabbage	Lp DL3	Plantaricin DL3	Disruption of pathogen cell wall and leakage of proteins	*P. aeruginosa, L. monocytogenes, Shewanella putrefaciens, Psychrobacter sp., S. aureus, B. cereus, Bacillus licheniformis, P. fuorescens*	Lv X, 2018
Olive	Lp NI326	Plantaricyclin A (PlcA)	n.i.	*Alicyclobacillus acidoterrestris, Lactococcus lactis spp., Lactobacillus bulgaricus UCC, Pediococcus inopinatus 1011*	Borrero J, 2018
Fermented stinky bean	Lp S0/7	Organic acids	Lowering cytoplasmic pH of target pathogens	*E. coli DMST4212, S. aureus DMST8840, B. cereus DMST5040, L. monocytogenes DMST17303*	Saelim K, 2017
Human breast milk	Lp WLPL04	EPSs	Inhibition of the biofilm formation or modification of the bacterial cell surfaces	*P. aeruginosa CMCC10104, E. coli O157:H7, S. Typhimurium* ATCC *13311, and S. aureus CMCC26003*	Liu Z, 2017
Shpek, bulgarian salami	Lp ST8Sh	Bacteriocin ST8SH (pediocin PA-1 family)	Pathogen’s cell lysis and intracellular material leakage	*L. monocytogenes Scott A, Enterococcus faecalis* ATCC *19433* *S. aureus*	Todorov SD, 2016 and 2017
Salami	Lp MBSa4	Plantaricin W	Bacteriostatic: electrostatic interactions with cytoplasmic membranes of bacteria, binds to the cell surface, but not killing effect	*L. monocytogenes,* S. aureus ATCC 25923, *Enterococcus hirae, Enterococcus faecium, L. innocua, L. welshimeri*	Barbosa MS, 2016
Yak yogurt	Lp Q7	Plantaricin Q7	n.i.	*Pseudomonas fluorescens AS1.1802, P. putida AS1.1819, P, aeruginosa CICC 21636, L. monocytogenes ATCC 19111, S. aureus, E. coli ATCC 25922, S. flexneri ATCC 12022, Shigella sonnei ATCC 25931, S. enterica serovar typhimurium ATCC 14028*	Liu H, 2016
Wine	Lp 105 Lp 106, Lp 107 Lp 119, Lp 32, Lp108	CFS (pH acid)	n.i.	*L. monocytogenes CECT 4032, E. coli O157:H7, S. Enteritidis CECT 409, S. aureus R1070, R1208, S1209, and S1220*	Arena MP, 2016
Suan-Tsai: chinese fermented cabbage	Lp JLA-9	Plantaricin JLA-9	Inhibited growth by preventing the establishment of oxidative metabolism and disrupting membrane integrity in germinating spores of *B. cereus*	*B. cereus, B. pumilus, B. megaterium, Bacillus coagulans, B. subtilis, Geobacillus stearothermophilus, Alicyclobacillus acidoterrestris, Paenibacillus polymyxa, C. difficile, C. perfringens, C. sporogenes, S. aureus, M. luteus, P. fluorescens, S. marcescens, E. coli, S. enteritidis, S. typhimurium, S. paratyphi A, S. paratyphi B, S. flexneri, Proteus mirabilis*	Zhao S, 2016
Kimchi	Lp K25	Plantaricin K25	Membrane surface disruption of the B. cereus cells, leakage and release of cellular contents	*B. cereus, L. monocytogenes NCTC 10890*	Wen LS, 2016
Dosa batter	Lp LD4	bacteriocin LD4	K^+^ ion efflux and pore-forming on membrane of M. luteus and E. coli cells	*M. luteus, S. aureus, E. coli* (urogenic), *P. aeruginosa, S. typhi, Vibrio sp., E. cloacae, E. faecium*	Kumar V, 2016
Meat	Lp KL-1	Plantaricin KL-1Y	Bactericidal activity without cell lysis	*B. cereus* JCM *2152T, S. enterica serovar Enteritidis DMST 17368, P. aeruginosa ATCC 15442, P. aeruginosa ATCC 9027, E. coli O157:H7, E. coli ATCC 8739, B. coagulans JCM 2257T, L. innocua ATCC 33090T, S. aureus TISTR 118*	Rumjuankiat K, 2015
Indonesian beef	Lp IIA-IA5	Plantaricin IIA-1A5	Loss of membrane integrity, release of proteinaceous and genetic materials	*S. aureus, Enteropathogenic E. coli K1, Shigella A33, Salmonella 38*	Sihombing DE, 2015 Arief II, 2015
Kefir grains	Lp YW32	EPSs	Concentration-dependent inhibitory effect on the biofilms’ formation	*E. coli O157, S. flexneri CMCC, S. aureus AC1, S. typhimurium S50333*	Wang J, 2015
Sheep-milk cheese	Lp U4	Plantaricin LpU4	Bacteriostatic mode of action and an enhanced activity at acidic pHs	*E. faecalis JH2-2, MRSA*	Milioni C, 2015
Koshu vineyard	Lp 510	Plantaricin Y	n.i.	*L. monocytogenes BCRC 14845*	Chen Y, 2014
Vaginal microbiota	Lp CMUL140	bacteriocin-like inhibitory substances (BLIS)	n.i.	*G. vaginalis CIP7074T, E. coli CIP103982, S. aureus ATCC 33862*	Al Kassaa I, 2014
‘Kanjika’ (ayurvedic rice-based fermented product)	Lp CFR 2194	Biosurfactants	Cell membrane lysis; antiadhesive activity	*E. coli ATCC 31705, E. coli MTCC 108, S. aureus F 722, Y. enterocolitica MTCC 859*	Madhu AN, 2014
mustard	Lp ZJ5	Plantaricin ZJ5	n.i.	*S. aureus CGMCC 1.128, L.* *plantarum, L. monocytogenes, B. subtilis, M. luteus, P. putida, E. coli, Shigella dysenteriae*	Song DF, 2014
Breast milk	Lp R315	EPSs	n.i.	*L. monocytogenes CMCC54007, S. aureus CGMCC26003, B. cereus ATCC 14579, S. typhimurium ATCC 1331, C. sakazakii ATCC 29544, S. sonnei ATCC 25931*	Li S, 2014
Fresh milk	Lp ZJ008	Plantaricin ZJ008	Bactericidal mode of action, pores formation in the surface of cell membrane but not cell lysis	*S. citreus LC5, S. carnosus LTH1502* *MRSA D48, S. epidermidis Z80, Micrococcus luteus 10209, L. monocytogenes LM1, E. coli DH5α, S. flexneri DSM4782*	Zhu X, 2014
Dairy	Lp HKN01	bacteriocin-like	n.i.	*E. coli (PTCC 1338), S. Typhimurium (ATCC 13311), K. pneumoniae (PTCC 1290)*	Sharafi H, 2013
Vegetable	Lp 163	Plantaricin 163	n.i.	*S. aureus, B. cereus, L. monocytogenes, B. pumilus, E. coli, P. aeruginosa, and P. fluorescens, M. luteus, L. thermophilus, L. rhamnosus*	Hu M, 2013
Meat	Lp BM-1	bacteriocin BM-1	Bactericidal mode of action without cell membrane lysis	*L. monocytogenes ATCC 54003, E. facealis AS 1.2984, L. pentosus ATCC 8041, L. plantarum F1, S. aureus ATCC6535, E. coli CDC85933, S. dysenteriae CMCC 51105 and S. enteritidis CMCC 50041*	Zhang H, 2013
-	Lp ATCC 10241	CFS	Prevents *P. aeruginosa* quorum-sensing; inhibition of biofilm formation; inhibited production of virulence factors (elastase, pyocyanin, rhamnolipids)	*P. aeruginosa*	Ramos AN, 2012
Papaya	Lp ST16Pa	bacteriocin ST16Pa	Bactericidal mode of action, cell lysis and enzyme-leakage	*L. innocua 2030C, L. sakei ATCC 15521, E. faecalis ATCC 19433*	Todorov, 2011
Thai dyspeptic patient	Lp B7	CFS (pH acid)	Inhibition of the pathogen’s urease activity and viability	*Helicobacter pylori ATCC 43504*	Sunanliganon C, 2012
Koumiss	Lp LB-B1	pediocin LB-B1	n.i.	*L. monocytogenes, Lactobacillus spp, Streptococcus spp, Enterococcus spp, Pediococcus spp, E. coli*	Xie Y, 2011

^1^ Lp: *L. plantarum*; ^2^ n.i., not investigated by the authors; ^3^ CFS: cell-free supernatants; ^4^ EPSs: Exopolysaccharides; ^5^ L-PLA: L-phenyl lactic acid; ^6^ SCFA: short-chain fatty acids; ^7^ LPB102: 2-(2-1 mino-1-hydroxyethoxy) ethyl 2-methylpropanoate; ^8^ PO: phenoloxidase.

**Table 2:** Probiotic *L. plantarum* strains with documented in vitro antiviral activity.

Isolation Niche	Strain Name	Type of Antiviral	Mechanism	Strong Antiviral Activity/ Virus Inhibited	Reference
Kimchi	Lp ^1^ NIBR97	Plantaricin 3 and 5	Lysis through envelope collapse	*HIV-based lentivirus, Influenza virus A/H3N2*	Kim SW, 2020
-	Lp ATCC LP299v	Metabolites	n.i.	*Rotavirus Wa*	Bernal SC, 2020
Animals faeces	Lp AA09a	CFS ^2^	n.i.	*Echovirus 7 (E7), E19*	Sunmola AA, 2019
Piglet faeces	Lp-1s	CFS	n.i.	*Transmissible gastroenteritis virus (TGEV)*	Wang K, 2019
Kimchi	Lp LRCC5310	EPSs ^3^	n.i.	*Human rotavirus (HRV)*	Kim K, 2018
Wine	Lp UNIFG30 Lp UNIFG121	CFS	n.i.	*Enterovirus Coxsackievirus B4*	Arena MP, 2018
Pig faeces	Lp 22F, 25F, 31F	CFS	n.i.	*Porcine epidemic diarrhoea virus (PEDV)*	Sirichokchatchawan W, 2018
Kimchi	Lp LBP-K10	Cyclic dipeptides	Conformational structures of cyclic dipeptides influence genes that cause viral infections	*Influenza A (H3N2) virus*	Kwak MK, 2013

^1^ Lp: *L. plantarum*; ^2^ CFS: cell-free supernatants; ^3^ EPSs: exopolysaccharides.

**Table 3:** *L. plantarum* strains with antimicrobial activity, whose probiotic and antipathogenic efficacy was tested in vivo (clinical and/or preclinical investigations).

Strain Name	Nature of Antimicrobial Postbiotic	Some Pathogens Inhibited	Application	Reference
Lp ^1^ 423	plantaricin 423	*L. monocytogenes EGDe*	Competitive exclusion of *L. monocytogenes EGDe* from the GIT of mice by plantaricin 423	van Zyl WF, 2019
Lp LMT1-48	SCFA ^2^ (hypothesised)	*E. cloacae*	Antiobesity effects in an *E. cloacae*-induced high-fat diet (HFD)-fed animal obesity model	Choi WJ, 2019
Lp ST8SH	Bacteriocin	*S. aureus*	Antibacterial activity in a rabbit model of femoral fracture with internal fixation	Xu Z, 2019
Lp SGLAB01	CFS ^3^	*V. parahaemolyticus*	Modulation of the immune system and increase shrimp resistance to *V. parahaemolyticus* infection	Chomwong S, 2018
Lp ATCC 8014	Bacteriocin	*S. aureus*	Control post-operative infection of mandibular fracture in mice model	Fu T, 2017
Lp HKN01	Bacteriocin-like	*E. coli (PTCC 1338), S. Typhimurium (ATCC 13311), K. pneumoniae (PTCC 1290)*	Recovery of *S. typhimurium-* infected BALB/c mice	Sharafi H, 2013
Lp B7	CFS (pH acid)	*H. pylori*	Attenuate *H. pylori*-induced gastric inflammation in rat	Sunanliganon C, 2012

^1^ Lp: *L. plantarum*; ^2^ SCFA: short-chain fatty acids; ^3^ CFS: cell-free supernatants.
